# Metabolism and Endocrine Disorders: What Wnt Wrong?

**DOI:** 10.3389/fendo.2022.887037

**Published:** 2022-05-06

**Authors:** Carolina N. Franco, May M. Noe, Lauren V. Albrecht

**Affiliations:** ^1^Department of Pharmaceutical Sciences, School of Pharmacy, University of California Irvine, Irvine, CA, United States; ^2^Department of Developmental and Cell Biology, School of Biological Sciences, University of California Irvine, Irvine, CA, United States

**Keywords:** methionine, SAM, arginine methylation, lysosomes, sclerostin, metabolomics

## Abstract

A fundamental question in cell biology underlies how nutrients are regenerated to maintain and renew tissues. Physiologically, the canonical Wnt signaling is a vital pathway for cell growth, tissue remodeling, and organ formation; pathologically, Wnt signaling contributes to the development of myriad human diseases such as cancer. Despite being the focus of intense research, how Wnt intersects with the metabolic networks to promote tissue growth and remodeling has remained mysterious. Our understanding of metabolism has been revolutionized by technological advances in the fields of chemical biology, metabolomics, and live microscopy that have now made it possible to visualize and manipulate metabolism in living cells and tissues. The application of these toolsets to innovative model systems have propelled the Wnt field into new realms at the forefront answering the most pressing paradigms of cell metabolism in health and disease states. Elucidating the basis of Wnt signaling and metabolism in a cell-type and tissue-specific manner will provide a powerful base of knowledge for both basic biomedical fields and clinician scientists, and has the promise to generate new, transformative therapies in disease and even processes of aging.

## Introduction

The goal of the present review is to introduce this new field of research linking Wnt signaling, cell metabolism, and endocrine disease. Canonical Wnt signaling has profound effects on cellular metabolism that occur on the timescale of hours, through ß-catenin gene transcription, and minutes, as Wnt ligands induce rapid regulation of cellular homeostasis prior to the transcriptional programs. This flurry of discoveries highlights the enormous potential of Wnt-targeted therapies and the current gaps in knowledge to be addressed before the links between Wnt and metabolism can be fully exploited.

## Wnt Signaling Pathway and Endocrine Systems

### Who Are the Key Players of the Canonical Wnt Pathway?

Canonical Wnt signaling is a fundamental pathway during embryogenesis and in adult tissues (1, 2). The non-canonical Wnt pathway regulates processes, such as cell polarity and directional movement, through Frizzled (Fz) interactions with Receptor Tyrosine Kinases (RTK) that activate non-ß-catenin effectors, such as calcium-calmodulin dependent kinase II, Protein Kinase C, sarcoma family kinase, heterotrimeric G proteins, and c-Jun N-terminal Kinase (3). The focus of this review will be the canonical Wnt signaling pathway.

**Figure 1:**
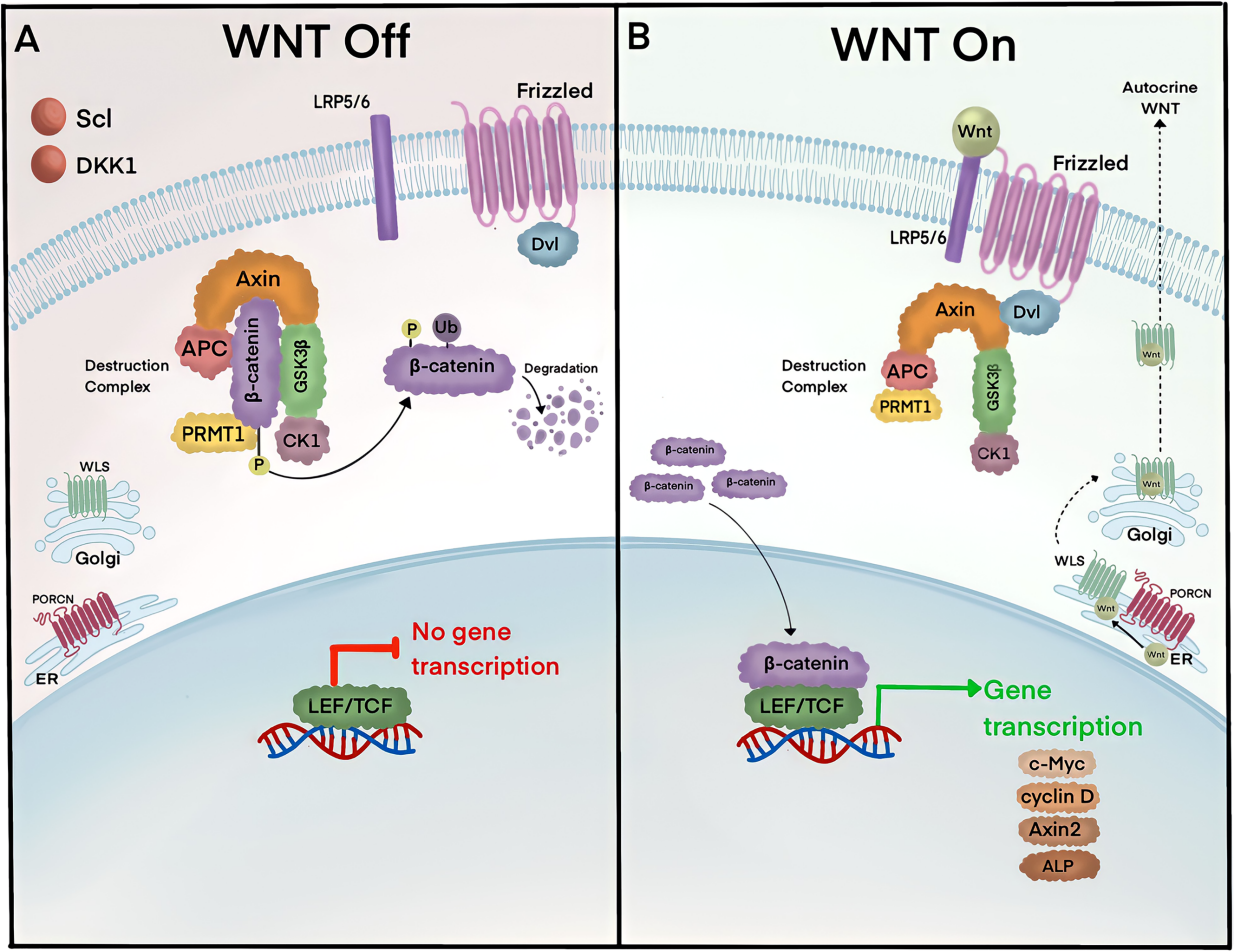
ß-catenin lies at the heart of the canonical Wnt signaling pathway. **(A)** In the absence of the Wnt ligands, Axin, APC, and GSK3 form the ß-catenin destruction complex. Axin serves as a scaffold for APC and GSK3, which bind and phosphorylate cytoplasmic ß-catenin, respectively. Phosphorylated ß-catenin is ubiquitinated by ß-TrCP and targeted for degradation in proteasomes. **(B)** Pathway activity is initiated by Wnt ligands that are secreted by neighboring cells. Wnt ligands are produced in the ER and become palmitoylated by Porcupine (PORCN) receptors, which in turn translocate Wnt ligands into the Golgi for endosomal exocytosis. Extracellular Wnt ligands bind to receptors Fz and LRP5/6 and trigger the removal of destruction complexes and Protein Arginine Methyltransferase 1 (PRMT1) from the cytosol to stabilize ß-catenin. Accumulated ß-catenin translocate into the nucleus and binds to LEF/TCF and induces gene transcription of targets such as c-Myc, cyclin D, Axin2, and alkaline phosphatase (ALP). Pathway activity is turned off by multiple mechanisms, including negative feedback inhibition whereby ß-catenin induces gene transcription of secreted Wnt inhibitors, sclerostin (Scl) and Dickopff 1 (Dkk1).

The canonical Wnt pathway is composed of a discrete set of proteins that lead to the transcriptional activity of ß-catenin (**Figure 1**). In the absence of Wnt activity, ß-catenin is continually degraded by a complex of proteins known as the destruction complex, composed of Axin, Disheveled (Dvl), Adenomatous Polyposis Coli (APC), Casein Kinase 1 (CK1), and Glycogen Synthase Kinase 3ß (GSK3ß) (4). GSK3ß specifically catalyzes phosphorylation on the N-terminus of ß-catenin allowing ubiquitin enzymes, ß-transducin repeat-containing E3 Ubiquitin protein ligase (ß-TrCP), to recognize and ubiquitinate residues for proteasomal degradation (5, 6). Upon pathway activation, Wnt ligands are secreted from neighboring cells and bind to the extracellular domains of Wnt receptor proteins, Low-Density Lipoprotein Receptor-Related Protein (LRP5/6) and Frizzled (Fz), which inhibit destruction complexes in the cytosol and stabilize ß-catenin (4). Accumulated ß-catenin translocate from the cytoplasm into the nucleus and induces the transcription of target genes such as the T-cell factor/lymphoid enhancer factor (TCF/LEF), Axin2, and Alkaline Phosphatase (ALP) (5, 7–13).

Destruction complexes are dynamically regulated through multiple mechanisms. Wnt signalosomes are protein complexes formed by destruction complex recruitment to the cytoplasmic face of Wnt receptors. Dynamic polymerization of Dvl increases avidity for Axin at LRP5/6, which then becomes phosphorylated and directly competes for GSK3ß (14–17). An alternate pathway employed by the cell removes GSK3ß through multi-vesicular bodies (MVBs) (18). In this model, Wnt ligand binding to receptors is followed by the rapid translocation of cytosolic GSK3ß into MVBs through microautophagy *via* endosomal sorting complexes required for transport (ESCRT)-dependent machinery (19, 20). More recently, biomolecular condensates were also revealed to be a strategy for destruction complex removal from ß-catenin (21). How these mechanisms and others coexist and why the cell would employ multiple approaches to regulate destruction complexes remains unknown. However, given the importance of the Wnt pathway across a diverse range of tissues and developmental stages, it is plausible that destruction complex regulation is ensured by the presence of multiple mechanisms. Alternatively, the use of one mechanism over another could confer precise spatiotemporal responses that activate ß-catenin with discrete kinetics, depending on the specific needs of the cell at that time.

The exquisite regulation of the Wnt pathway is also orchestrated at the level of membrane receptors by secreted Wnt factors. ß-catenin transcriptional activity stimulates a negative-feedback loop through the expression of Ring Finger protein 43 (RNF43) and Zinc and Ring Finger protein 3 (ZNRF3), which ubiquitinate Wnt receptors to remove them *via* endocytosis (22, 23). Secreted proteins R-Spondin (RSPO) bind to Leucine-rich repeat-containing G-protein-coupled receptors (LGR4-5), which reside in the plasma membrane and auto-ubiquitinate E3-polubiquitin ligases (RNF43/ZNRF3) for endocytic removal (24, 25). Thus, R-Spondin ligands represent potent canonical Wnt activators by increasing the abundance of Fz and LRP5/6 receptors. In contrast, secreted Wnt inhibitors such as Dickkopf1 (Dkk1) (26) and sclerostin (Scl) bind to LRP6 and prevent key interactions with Fz (27). Together, this complex regulatory system confers spatial specificity to the Wnt pathway in different cell types and biological contexts using the same core set of molecules.

### Canonical Wnt Signaling in Endocrine Organ Physiology

**Figure 2:**
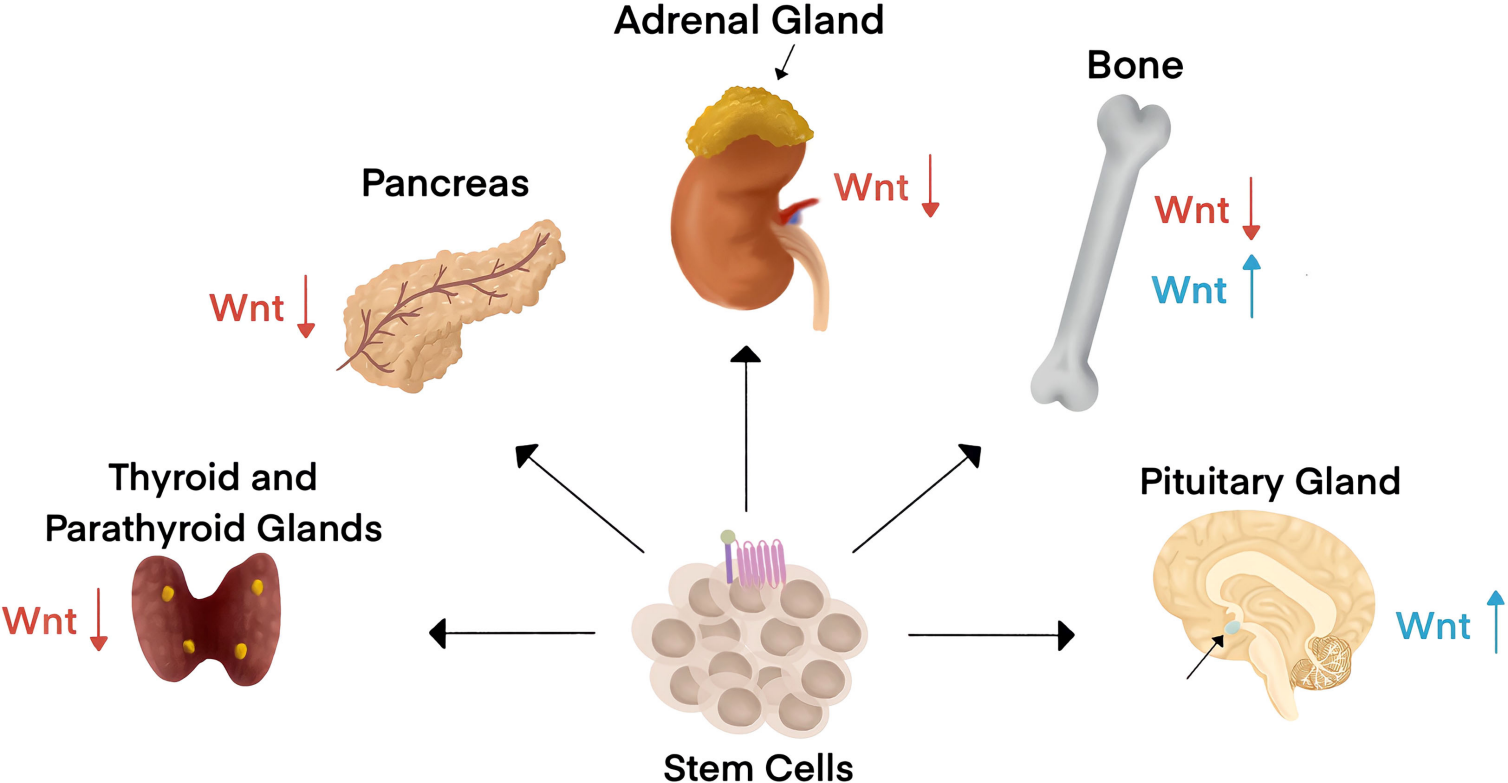
Canonical Wnt signaling impacts endocrine organ physiology and pathology. Wnt is the stem cell niche signal across endocrine organs. Endocrine systems are designated by the criteria of hormone secretion into the blood to affect changes in distant organs. Wnt signaling has been associated with endocrine disorders in the thyroid, parathyroid, pancreas, adrenal gland, bone, and the pituitary gland. Wnt signaling activity is typically downregulated (red arrows) in diseases associated with all endocrine organs except for the pituitary gland and in some bone-associated endocrine disorders, where Wnt signaling activity is upregulated (blue arrows).

The endocrine system consists of a network of glands that release hormones and control body functions. Canonical Wnt signaling has pleiotropic roles across endocrine systems during embryogenesis and in adults to regulate tissue homeostasis and remodeling (**Figure 2**). Canonical Wnt is the stem cell niche signal across endocrine organs. For example, Wnt promotes the continual renewal of mesenchymal stem cells and is essential for all mesenchymal tissues. Wnt signaling drives adrenocortical progenitors and contribute to the maintenance of adrenal homeostasis (28). Contrary to the role of Wnt signaling in epithelial stem cells, single-cell RNA sequencing recently revealed that Wnt signaling is low in endocrine progenitor stem cells (29).

Wnt signaling is a fundamental pathway in regulating bone tissues. Bone remodeling is orchestrated by the dynamic interplay of three types of bone cells. Osteoblasts are responsible for bone formation while osteoclasts degrade the bone matrix. Osteocytes represent 90% of bone cells and reside within the bone mineralized matrix (30). While these cells were originally thought to operate simply as placeholders within the bone, we now know that osteocytes are highly active and have critical functions in bone mass regulation and whole-body homeostasis. Notably, osteocytes sense mechanical forces and translate signals into chemical cues. Importantly, osteocytes have even emerged to be endocrine organs themselves as they secrete factors that regulate the activities of neighboring bone cells and even within distant organs.

Osteocyte secreted factors and the multitude of functions have been recently reviewed (31). Osteocalcin is secreted by mature osteoblasts and osteocytes to regulate a dynamic interplay between the bone and pancreas; Osteocalcin knockout mice have impaired glucose tolerance, lowered insulin secretion, and became obese (32). It is interesting to note that Wnt signaling contributes to islet development and function and regulates the production of insulin in ß cells (29).

Fibroblast growth factor 23 (FGF23) is an osteocyte secreted factor that suppresses phosphate reabsorption and vitamin D synthesis in the kidney. The pathophysiological consequences of elevated FGF23 levels involve kidney and bone disease, among others (30, 33). FGF23 has been reported to be increased in Chronic Kidney Disease (CKD) patients that also have elevated sclerostin-expressing osteocytes, highlighting the potential links between Wnt signaling and FGF23 (33, 34). Wnt signaling has well-established roles in promoting kidney organogenesis, repair, and regeneration (35). The discovery that FGF23 is linked to canonical Wnt signaling supports a strong rationale to understand the Wnt-driven signaling in CKD.

Adipose tissues integrate whole-body glucose homeostasis and energy balance. Autocrine and paracrine signals from Wnt ligands are famous for their pro-growth roles during embryogenesis and in cancer. This is in stark contrast to Wnt activities within adipocytes, whereby Wnt ligands function as key inhibitors of adipogenesis (36). LRP5 is essential for glucose-induced secretion and cholesterol metabolism (37). In preadipocytes, ß-catenin downregulates the nuclear hormone receptor peroxisome proliferator-activated receptor (PPARγ) and CCAAT/enhancer binding proteins (C/EBPα) and shifts differentiation towards osteoblastic and immune cell lineages (38, 39). The logic of this seemingly opposing role of Wnt signaling is underscored by conditions of limited vasculature nutritional support. For example, hypoxia increases Wnt signaling and subsequently suppresses adipogenesis in neighboring regions to conserve metabolism. Further, increased levels of circulating RSPO2 increases adipose tissue hypertrophy and insulin resistance in mouse models of obesity (40).

### Wnt Signaling in Endocrine Pathology

Endocrine disorders come in all shapes and sizes. Overproduction of hormones by a gland leads to hormonal imbalance-driving disorders such as diabetes, acromegaly, and bone loss in chronic kidney disease. Underproduction of hormones is the basis of disorders such as hypothyroidism, hypopituitarism, and growth hormone deficiency. The roles of Wnt signaling in these contexts have recently come to light.

### Genetic Mutations and Pathway Dysregulation

Aberrant Wnt signaling contributes to several endocrine disorders. For example, diseases characterized by altered hormonal production in adrenal and thyroid glands have been linked to low Wnt activity (**Table 1**). Pituitary glands are the size of a pea but not to be taken lightly, as overactive growth hormone secretion results in gigantism; loss of function mutations in Wnt inhibitors sclerostin and Dkk1 were identified in acromegaly patients (57).

**Table 1:** Wnt Signaling is Misregulated in Disorders Stemming from Endocrine Systems.

Protein	Genetics	Wnt activity	Condition	Organ System	Reference(s)
Dkk1	Increased*	Low	Gaucher’s**	NA	(41)
	Increased	Low	Type 1 Diabetes	Pancreas	(42)
	Increased*	Low	Hypothyroidism	Thyroid	(43)
LGR4	Missense mutation	High	Obesity & Metabolic Disorder**	NA	(44)
LRP4/5/6	Missense mutation	Low	Metabolic syndrome & Osteoporosis	Pancreas & Bone	(45)
	Polymorphism	Low	Type I Diabetes	Pancreas	(46)
	Loss-of-function	Low	Osteoporosis	Bone	(8)
	Gain-of-function	High	Dense bones, Osteosclerosis & Less insulin resistance	Bone	(47–51)
Sclerostin	Increased*	Low	Cushing’s	Adrenal gland	(52)
	Loss-of-function	High	Dense bones	Bone	(53–55)
	Increased	Low	Type 1 Diabetes	Pancreas	(42)
	Increased*	Low	Hyperparathyroidism/Hyperthyroidism	Thyroid gland	(43, 56)
	Increased*	Low	Hypothyroidism	Thyroid gland	(43)
	Decreased*	High	Acromegaly	Pituitary gland	(57)
SFRP2	Decreased*	High	Cushing’s	Adrenal gland	(58)
TCF7L2	Polymorphism		Type II Diabetes	Pancreas	(59, 60)
Wnt Ligand	Mutation	Low	Osteoporosis	Bone	(61)
	Loss-of-function & Missense	Low	Bone loss & Osteogenesis Imperfecta	Bone	(62–65)
	Loss-of-function	Low	Addison’s	Adrenal gland	(66)
	Polymorphism	High	Type II Diabetes	Pancreas	(67)
	Increased*	High	Obesity**	NA	(67)

*No genetic mutations reported; down and upregulation of protein levels.

**No endocrine organs.

Wnt signaling is an essential regulator of bone; genetic mutations in the Wnt pathway proteins lead to either high or low bone density disorders. Loss-of-function mutations in sclerostin lead to pathologically dense bones in diseases such as osteosclerosis (53–55). LRP5/6 mutations can lead to altered bone remodeling characterized by high bone density in addition to osteoporosis. Loss-of-function LRP5/6 mutations were identified in osteoporosis pseudoglioma (OPPG) patients, a disease characterized by thinning of the bones and impaired vision (8). Conversely, gain-of-function mutations in LRP5/6 lead to high bone mass (47–50). Interestingly, in a mouse model of insulin-deficient diabetes, gain-of-function LRP5 mutations improved bone mass and delayed hyperglycemia (51). Osteogenesis Imperfecta (OI) is a genetic bone disease caused by mutations in Wnt1 that is characterized by an early onset of osteoporosis with increased bone fragility and reduced bone mass (61–64).

Cushing’s syndrome is characterized by elevated serum steroid hormone cortisol that is caused by a tumor in the pituitary gland that releases a hormone known as Adrenocorticotropic Hormone (ACTH). The role of Wnt signaling in Cushing’s syndrome was underscored by reports showing that patients have increased rates of fracture and elevated levels of sclerostin in circulation (52; (68). In contrast, *in vitro* studies using ACTH secreting glands isolated from Cushing’s patients displayed a downregulation of the Wnt inhibitor, secreted frizzed related protein 2 (SFRP2), to promote pituitary corticotroph adenoma (58). Conversely, Addison’s disease is characterized by adrenal insufficiency, or the inability to produce adequate amounts of steroid hormone cortisol. Loss-of-function Wnt4 mutations led to impaired steroid production by decreasing *Cyp11B2* expression, the gene encoding for aldosterone synthase (66). Together, these studies highlight a complex interplay between secreted Wnt regulators, organ systems, and disease pathology ripe for future investigations.

Wnt pathway inhibitors link bone and thyroid signaling. Hyperthyroidism is driven by the upregulation of thyroxine (T4), which increases physiological metabolic rate and leads to weight loss (69). Mouse models of hyperthyroidism demonstrated an upregulation of sclerostin in serum with reduced trabecular and cortical bone density (43). Interestingly, sclerostin levels were also higher in hypothyroid mice that presented with high bone density. Together, these findings illustrate a complex interplay between Wnt activity and thyroid signaling.

Diabetes is a chronic condition that affects how the body converts food into energy. Obesity is a major concern of public health that is a result of genetic and environmental factors. In Type 1 Diabetes (T1D), an autoimmune attack renders pancreatic islet cells unable to produce insulin, increasing blood sugar. Type 2 Diabetes (T2D) is characterized by insulin resistance, a condition in which the body does not respond appropriately to insulin. Interestingly, genetic mutations in the Wnt pathway proteins were identified in T2D and obesity (70–72). In T2D, genetic mutations in TCF family member TCF7L2 (formerly TCF4) contributed to adipocyte hypertrophy and insulin resistance (59, 60, 73, 74). Additional mutations in T2D were identified in Wnt5b (67) and in LRP6, which resulted in hyperlipidemia and impaired glucose metabolism (45). LGR4 and Wnt5b mutations have also been linked to increased activity of the Wnt pathway in metabolic disease (44, 67). While it is difficult to specifically tie mutations to metabolic syndrome in T2D and obesity, these genetic mutations highlight a potential link between canonical Wnt and the onset or even susceptibility to these diseases.

GSK3 was originally discovered through its fundamental role in regulating glycogen synthase (75). We now know that is an important player of glucose metabolism and use of GSK3 inhibitors have been explored as a potential therapeutic window for diabetic patients. In this context, GSK3 inhibitors operate decrease glucose levels by restoring glycogen synthase activity. Beyond this, GSK3 facilitates proteasomal degradation of many substrates whereby phosphorylation cascades trigger subsequent ubiquitination. It is through this molecular mechanism that GSK3 has been linked to a variety of metabolic pathways. GSK3 phosphorylates IRS-1 to spark ubiquitination and degradation, which subsequently leads to insulin resistance (76). GSK3 has also been implicated in cellular nutrient-sensing properties through the regulation of mammalian target of rapamycin (mTOR) and tuberous sclerosis complex 2 (TSC2) (77). Specifically, GSK3 suppresses glucose uptake by the Glut1 transporter *via* mTOR and TSC2 phosphorylation (77). In the context of cancer cell metabolism, it was reported that GSK3 inhibition releases c-MYC and c-JUN from proteasomal degradation and as a result increases the expression of glutaminase and the levels of glutamate (78). In hepatocellular carcinoma models, GSK3 inhibited cells were evaluated by metabolomics analyses and revealed an increase in specific amino acids such as Arginine, Lysine, Methionine and Glutamine (79). Altogether, the roles of GSK3 signaling cascades in metabolism will expand with the application of metabolomic analyses and single cell RNAseq to various contexts and diseases.

## Wnt Signaling and Cellular Metabolism

Studies of Wnt signaling and the regulation of cellular metabolism have predominantly focused on elucidating processes of growth during development and in cancer. Recent work highlights that similar reprogramming of metabolic pathways drive the progression of endocrine disease, suggesting that Wnt signaling may similarly be implicated. In this section, we will compare how Wnt signaling regulates metabolic pathways in cancer, which may contribute to endocrine disease.

### Cancer Cell Metabolism

**Figure 3:**
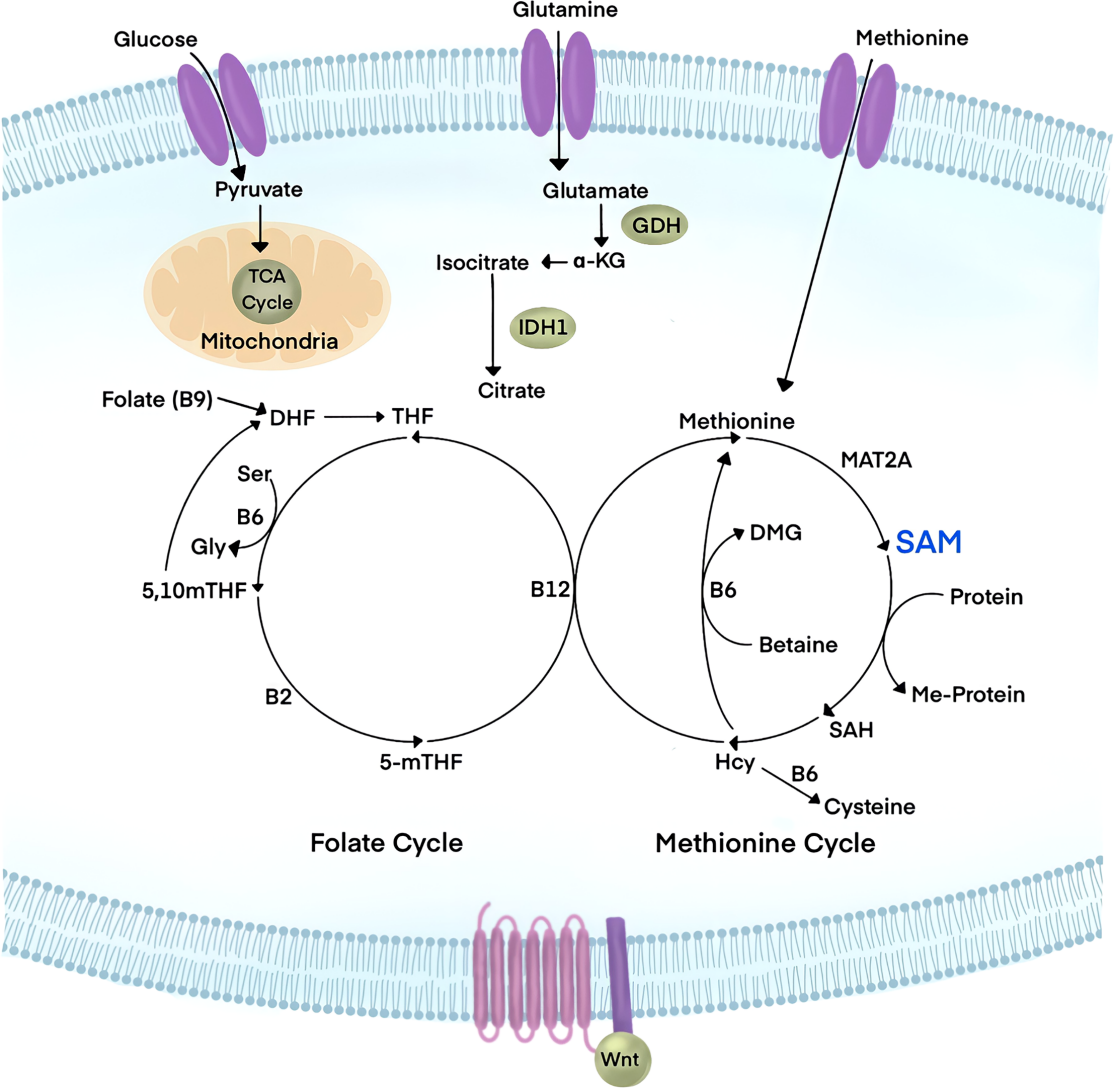
Wnt signaling has emerged to be a major player in the regulation of cellular metabolism. Influx of glucose, glutamine, and Methionine metabolites have been linked to canonical Wnt signaling. Glucose is used by cells for energy metabolism in glycolysis and the Tricarboxylic Acid cycle (TCA), yielding pyruvate, ATP, and NADH. Glutamine is avidly consumed in cancer and leads to the reduction of downstream metabolites such as α-ketoglutarate (α-KG). Methionine is an essential amino acid that feeds the Methionine cycle to produce S-Adenosylmethionine (SAM), the universal methyl-donor of the cell. Upon substrate methylation, SAM is converted to S-Adenosylhomocysteine (SAH), which subsequently generates homocysteine (Hcy). The Methionine cycle is linked to the folate cycle in a larger metabolic pathway known as One Carbon Metabolism. Targeting the PRMT1 cofactor, SAM, through metabolic interference could tune Wnt signaling responses.

Cellular metabolism during embryonic development is geared towards proliferation and the production of biomass (**Figure 3**). Cancer cells hijack physiological growth mechanisms to fuel the vast amount of nutrients required to support tumorigenesis (80). The principal source of carbon that is consumed in cells is glucose. Glycolysis, the catabolism of glucose, produces carbon in the Tricarboxylic Acid (TCA) cycle and supports macromolecule biosynthesis. The TCA cycle produces cellular energy, ATP, and NADH for oxidative phosphorylation (OXPHOS) *via* the electron transport chain (81).

Growth factors stimulate glucose uptake to promote growth processes in physiological conditions. Cancer cells can adapt to harsh microenvironments to meet the high nutritional demands for aberrant proliferation. Normally under hypoxic conditions, cells are forced to use glycolysis to generate ATP. This results in a build-up of lactic acid. Cancer cells preferentially use glycolysis rather than the TCA cycle to generate energy, independent of oxygen levels in what is known as the “Warburg Effect”. A hallmark study revealed that canonical Wnt signaling drives the Warburg Effect to promote colorectal carcinoma (CRC) (82). Since this landmark discovery, Wnt signaling has been found to drive multiple metabolic pathways that are linked to cancer cell metabolism. In CRC, cancer cells utilize glutamine at exaggerated rates to promote growth and survival, which depletes downstream metabolites. It was recently reported that oncogenic Wnt signaling is tightly linked to the homeostasis of glutamine and α-ketoglutarate (α-KG); supplementation of α-KG was sufficient to downregulate Wnt activity (83). Loss-of-function Axin mutations are among the most frequent drivers of hepatocellular carcinoma (HCC) and have been linked to multiple metabolic pathways. Wnt signaling increased unsaturated fatty acyl groups in phospholipids in HCC to promote lipid metabolism during tumorigenesis (84). Finally, another adaptative response to the rapid cell cancer growth is the relative hypoxic state within the tumor microenvironment, which generates reactive oxygen species (ROS) (85). Additionally, hyperactive Wnt signaling in cancer was shown to contribute to elevated ROS levels (86, 87). Future studies to identify the relative metabolic vulnerabilities of different cancers could lead to new therapeutic points of intervention.

### Metabolic Landscape of Endocrine Disease Versus Cancer

Beyond these tantalizing reports linking Wnt signaling and cellular metabolic reprogramming of cancer, canonical Wnt also regulates tissues outside of cancer. For example, bone formation is driven by a subset of cells lining the calcified bone matrix known as osteoblasts. Wnt signaling is a well-established regulator of osteoblastic activity that leads to new calcified bone tissues. Interestingly, similar to its role in cancer, Wnt activity was found to increase glycolic enzymes through the Warburg Effect to generate energy in osteoblasts. This finding was shown using the LRP5 knockout mouse and resulted in low bone mass and low circulating serum lactate levels, which were linked to reduced mTOR activity and low glycolysis (88). Additionally, this report investigates the dramatic effects of Wnt stimulation after only 20 minutes of pathway activation. This report and others reveal the emerging importance of Wnt as a rapid regulator of cellular metabolism as pathway activity orchestrates cellular responses, prior to the transcriptional programs that occur after hours. This is particularly important for the fields investigating the links between Wnt and metabolism as cellular changes in metabolism occur within seconds (89).

The human body contains 6,500 discrete metabolites (90, 91). Wnt signaling has been linked to regulation of obscure metabolites in cellular pathways related to the insulin sensitivity of pancreatic ß cells (92). Metabolomics-based mass spectrometry revealed that two metabolites, two amino adipic acid (2-AAA) and fatty acid furan metabolite 3-carboxy-4-methyl-5-propyl-2-furanpropanoic acid (CNPF), were associated with disease risk in T2D (93). This mechanism was regulated by the NADP^+^-dependent isocitrate dehydrogenase (IDH1/2), a key TCA enzyme that converts isocitrate to α-KG. In myeloid malignancies and solid tumors, mutations in IDH1/2 changes the activity to generate 2-Hydroxyglutarate (2-HG), which competes with α-KG-dependent processes (94). Interestingly, in rat insulinoma-derived cell lines, IDH1/2 regulates glucose-stimulated insulin secretion. α-KG generated by IDH1/2 serves as a building block for glutathione, which is a key player in insulin release (93). Given the recent discovery that Wnt and α-KG are linked and contribute to CRC, it is tempting to speculate whether this metabolic regulatory pathway can be harnessed for developing therapeutics. Collectively, elucidating how each discrete metabolite is regulated in health and disease states could offer key insight into systemic body metabolism regulation and how distinct pathways might be targetable for precision medicine.

### Post-Translational Modifications (PTMs) and Metabolite Fluxes in Endocrine Disease

**Figure 4:**
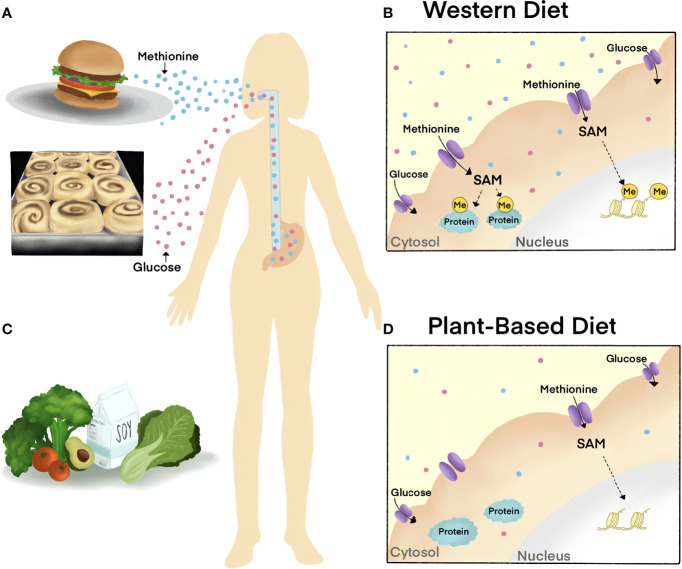
The impact of diet on cellular metabolite abundance and molecular signaling cascades. **(A, B)** Western diets are rich in Methionine and sugar, metabolites that are related to the progression of disease. Excessive dietary Methionine contributes to elevated levels of substrate methylation in the nucleus (histone, yellow) and cytoplasm (proteins, blue) as SAM is the universal methyl-donor of the cell. **(C, D)** Diets low in Methionine, such as plant-based diets, decrease cellular methylation by reducing SAM. The World Health Organization recommends a healthy diet that is low in salt and sugar, high in fruits and vegetables, and that incorporates unsaturated fats (such as in avocado) over saturated fats (such as in fatty meat).

How the Wnt signaling pathway uses one common set of molecules to generate vastly different biological functions remains a mystery. Post-translational modifications (PTMs) provide specialized details to modulate Wnt responses. Beyond the well-established roles of protein phosphorylation, we now know there are a variety of modifications that are central to regulating the Wnt pathway. Notably, many of these modifications require cofactors that are sensitive to changes in nutrient availability based on diet (**Figure 4**). This knowledge has only recently come to light as it was a product of the technological advances in metabolomics sensitivity. Importantly, how this novel concept relates to the progression of disease states and can be exploited for developing new therapies has been underscored by multiple enticing reports.

The cofactor for protein phosphorylation is ATP, a metabolite that is present on the millimolar range. Given this, the excessive cellular concentrations of ATP will not limit kinase activity and downstream signaling cascades, with the exception of AMPK. Enzymes catalyzing protein methylation and acetylation use S-Adenosylmethionine (SAM) and acetyl-coA as cofactors, respectively. In contrast to protein phosphorylation, the intracellular concentrations of SAM and acetyl-coA relative to the Michaelis constant (K_m_) render protein methylation and acetylation modifications sensitive to nutrient levels (95); Further, protein methylation and acetylation modifications are catalyzed on a slower timescale (95). Thus, the perturbation of certain metabolites in circulation influences the levels of cofactors that are available for enzymatic activity and cell signaling.

Protein methylation was recently identified to be required for the canonical Wnt pathway (96). Protein methylation of lysine and arginine residues use SAM as the methyl-donor and methylation removal is catalyzed by demethylase enzymes that use α-KG as a cofactor. Protein Arginine methylation is catalyzed by nine different enzymes known as Protein Arginine Methyltransferases (PRMTs) that can promote either symmetric or asymmetric dimethylarginine modifications. PRMT1 is responsible for catalyzing 85% of asymmetric dimethylarginine modifications on proteins and has been implicated in several human diseases such as Wnt -driven cancers. PRMT1 inhibitors have passed clinical trials and emerged as a novel target for the treatment of B-cell lymphoma (97).

Methionine restriction has been shown to alter protein methylation activity through PRMT activity. Methylthioadenosine phosphorylase (MTAP), a recently identified tumor suppressor, was shown to render cancer cells sensitive to methionine restriction by lowering SAM levels (98, 99). Similarly, approaches to reduce SAM levels by methionine restriction or methotrexate treatment were sufficient to decrease Wnt signaling in cultured cell models of cancer. PRMT1 was discovered to be a key regulator of the rapid cellular responses to Wnt signaling and for the removal of GSK3 from cytosolic ß-catenin (100). The loss of PRMT1 or disruption of SAM *via* methionine deprivation decreased Wnt transcriptional activity and could offer a novel point of intervention for Wnt-driven diseases (96). Further, Wnt ligand binding recruits PRMT1 to destruction complex protein GSK3 and leads to protein methylation on cytosolic substrates that are translocated into lysosomes to promote ß-catenin (96). These reports further the novel concept that Wnt rapidly tunes cellular metabolism as is found with Wnt/Stabilization of Proteins (Wnt/STOP) (18, 101).

### Lysosomes in Genetic Diseases

Amino acids are the building blocks of proteins. Protein catabolism also restores amino acids in cells through degradation in proteasomes or lysosomes. Lysosomes were originally thought to be simply the trash can of the cell. We now know that lysosomes are a central signaling hub and that their function is coopted during the progression of myriad diseases (102).

Lysosomal storage disorders (LSDs) are a group of rare inherited diseases that are characterized by dysfunctional lysosomes and are associated with hormonal imbalances and metabolic disorders. One of the most prevalent LSDs is Gaucher’s disease (GD) (103). GD is caused by mutations in the *GBA* gene that encodes for Glucocerebrosidase (GCase) enzyme, which breaks down lipids inside the lysosome. Patients present with nutritional depletion, hypermetabolic states, insulin resistance, and hypoadiponectinemia. Recent advances in the fields studying LSDs have been made through the application of induced pluripotent stem cell (iPSC) models. In iPSC GD models, low Wnt signaling was linked to higher levels of Dkk1. Similarly, low Wnt signaling activity was also reported in iPSC models of Niemann-Pick disease, an LSD that causes dysregulated lipid and cholesterol metabolism (104). In Niemann-Pick cells, low Wnt signaling was attributed to disordered lipid membranes that prevent Wnt ligand receptor binding (105). These reports raise the possibility that reengaging the Wnt pathway through monoclonal-based treatments may be beneficial for LSDs.

### Lysosomes in Cancer

Protein catabolism is usurped to reprogram tumor cell metabolism. Lysosomes have emerged as an important target in cancer as they replenish nutrients that enable growth. Recent work highlights a central role for the Wnt signaling pathway in regulating lysosomal activity. First, Wnt ligand stimulation rapidly replenished nutrients by activating lysosomes (96). Second, in the context of colorectal and hepatocellular carcinomas, the loss of tumor suppressor proteins APC and Axin similarly resulted in increased lysosomal metabolism, which fueled nutrient acquisition and aberrant growth (79, 96, 106, 107). In these cases, Wnt signaling drove macropinocytosis, a nonreceptor-mediated endocytic process to incorporate extracellular proteins into the cell for protein catabolism in lysosomes. Specific amino acids were increased by Wnt ligands after one hour of treatment. This raises the possibility that cells sense nutrient status of surrounding environments and activate Wnt signaling to replenish discrete sets of growth-promoting metabolites. Altogether, lysosomes represent a novel target for designing interventions across Wnt-driven cancers.

## Organ Crosstalk

### The Intersection of Bone and Kidney

**Figure 5:**
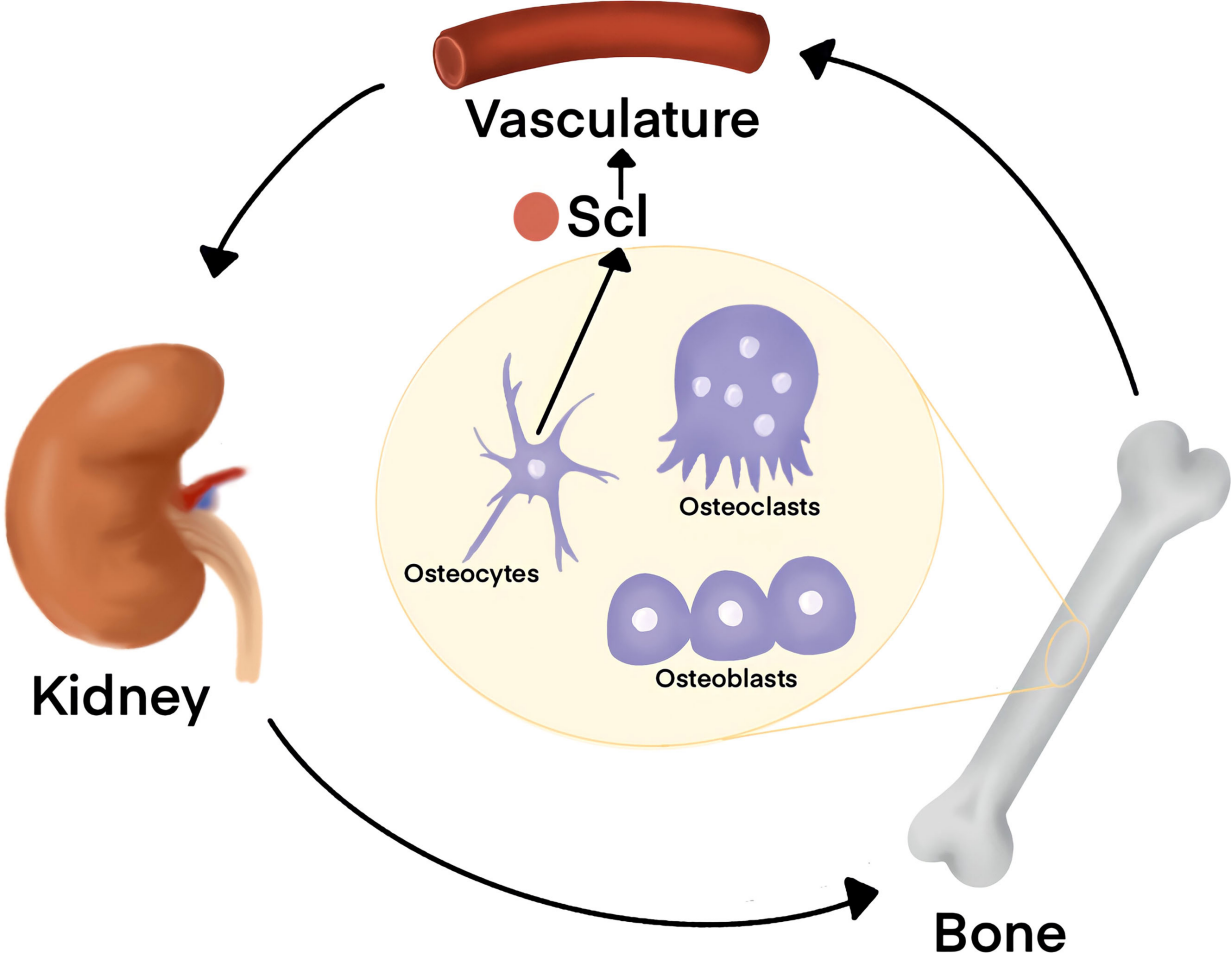
Organ crosstalk between bone and kidney is tightly regulated by Wnt signaling. Kidneys sense the levels of serum metabolites such as Phosphate and Calcium. When Calcium levels are low, Parathyroid Hormone (PTH) is released from the parathyroid, located on the posterior aspect of the thyroid gland on the bilateral superior and inferior poles, and orchestrates bone remodeling for metabolic homeostasis. Sclerostin (Scl) is secreted from osteocytes and antagonizes Wnt signaling in osteoblasts to decrease bone formation rates. In chronic kidney disease (CKD), elevated Scl in circulation is associated with worse patient outcome. Further, elevated serum Scl was even reported to occur prior to renal disease onset, suggesting that factors secreted from the bone may drive CKD.

Sclerostin is a secreted Wnt inhibitor that has emerged in the progression of metabolic disease and endocrine dysfunction. Normally, sclerostin is secreted from osteocytes, bone cells that are embedded within the bone matrix, and inhibits Wnt signaling in osteoblasts (55). sclerostin accumulates in the serum of patients with chronic kidney disease (CKD) and was associated with bone disease (108). In physiological conditions, bone and kidney crosstalk is orchestrated by the parathyroid gland (**Figure 5**). Healthy kidneys filter half a cup of blood every minute to remove waste and to assess the levels of circulating metabolites such as calcium, sodium, phosphorus, and potassium. When calcium levels are low, parathyroid hormone (PTH) secretion from the parathyroid gland sparks the activity of osteoclasts that degrade bone in a process known as resorption (109, 110). Bone resorption releases free calcium until systemic metabolic homeostasis is reached and PTH levels decrease. In CKD, elevated levels of PTH persist and result in pathologic bone loss and Mineral and Bone Disorder (CKD-MBD) (108). Interestingly, elevated sclerostin was even reported in patients prior to renal disease, suggesting that bone-secreted factors are upstream drivers in CKD and bone disease. Outside of CKD-MBD, sclerostin levels were also increased in T1D and played a role in the misregulation of glucose metabolism (51). These cases highlight the fact that aberrant Wnt activity in metabolic disease can stem from genetic mutations or through unknown causes. Monoclonal antibody-based therapies targeting sclerostin have been developed (Romosozumab) and were successful in restoring bone formation rates of elderly osteoporotic patients (111–113). The association between elevated sclerostin and disease progression suggests a potential for use of Scl-based therapies that extends beyond age-related pathologic bone loss (114).

### Diet-Based Intervention and Whole-Body Metabolic Disease

New strategies for disease management of obesity, diabetes, and cancer are emerging in the clinic. Caloric restriction delays aging processes and rejuvenates tissues (115, 116) and involves a 50% reduction of total caloric intake (117). Thus, one of the biggest challenges lies in the unrealistic expectations of patient adherence to strict modifications for prolonged periods of time. Ketogenic diets that are rich in fat and low in carbohydrates lead to a 30% reduction in blood glucose, and have been attributed to the reduction of circulating insulin and decreased mTORC1 signaling, and potentially oxidative stress (118). Monitoring how diet and systemic metabolism alter metabolism on the single cell level is now possible and opens a future for developing specific diets to treat disease states by simply removing one metabolite at a time.

The restriction of specific amino acids could help improve therapies in cancer. Many tumors rely on nutrients supplied by the host’s diet and can even become dependent on a single amino acid (119–121). Essential amino acids can only be obtained from diet, offering a unique opportunity to target the tumor microenvironment. Asparagine is involved in cancer proliferation by driving invasion and metastasis, and asparagine-restrictive diets that reduce consumption of asparagine-rich foods, like asparagus, reduce metastasis in mouse models of breast cancer (122, 123). Serine is the second most used non-essential amino acid in the human proteome (124). Upregulation of serine metabolism in cancer contributes to 50% of glutamine anaplerosis to α-KG in the TCA cycle (120). Further, growth was effectively inhibited by serine deprivation in CRC tumors driven by the loss of APC (124). Thus, elucidating which cancers are dependent on specific nutrients will provide key details that enable clinicians to prescribe dietary regimens that complement chemotherapeutic interventions.

Methionine is an essential amino acid obtained from specific foods, such as Brazil nuts and red meat. It is essential for methylation as it generates SAM, the universal methyl-donor of the cell, during one-carbon metabolism (80). Long-term Methionine restriction has been linked to lifespan extension. The effects of Methionine restriction on whole-body metabolism occur after only one week in mouse models of sarcoma, glioma, melanoma, prostate, colorectal, and breast cancer. In pre-clinical trials, short-term Methionine deprivation enhanced efficacy of chemotherapeutic intervention (121). These reports highlight the role of elevated methylation in cancer, yet the precise target of methylation modifications that are sensitive to Methionine-restricted diets are unclear. Interestingly, Wnt signaling was reported to act as the growth factor pathway for sensing cellular Methionine levels; the reduction of Methionine decreased transcriptional Wnt activity. The findings from this report further suggest that Wnt signaling acts as a regulator of the one-carbon pathway and Methionine metabolism to promote growth (100). There are still no clear guidelines of dietary recommendations for cancer patients as there is an unmet clinical need for understanding the molecular basis regulating these mechanisms and how the metabolic requirements for specific nutrients vary across different cancers.

Advances in the sensitivity of metabolomics-based mass spectrometry have revolutionized the global understanding of metabolism and have been active in the world of cancer cell biology. Though these discoveries have been groundbreaking, these studies have largely been limited as they have been conducted *in vitro*, and do not accurately recapitulate whole-body metabolism. The use of stable isotope-labels in disease animal models have recently been used to map the metabolic fates of nutrients exchanged between organs *in vivo* (125). Similarly, Faubert et al. developed methods to monitor metabolism in human patients and even in patient tumors through isotopic labeling of nutrients (126). These techniques of metabolic fluxes *in vivo* will likely provide paradigm-shifting insight into the fundamental links between diet, health, and disease that answer mysteries that have plagued mankind.

## Future Perspectives

The future is bright, particularly in light of several key technological advances in the field of metabolism. In our view, the key outstanding questions to fully understanding how the Wnt pathway orchestrates metabolism are clear. Addressing these gaps in knowledge could remove existing roadblocks of Wnt-targeted therapeutics.

### The Roles of Wnt That Extend Beyond Transcription

Wnt signalling through transcriptional activity has been well documented. However, emerging work highlights a clear role for Wnt signalling in the rapid regulation of cellular processes such as metabolic control (127, 128). This is logical given the fundamental nature of Wnt as a pro-growth signal that requires biomass and fresh metabolites quickly. Indeed, Wnt signalling promotes protein degradation in lysosomes as a means to refresh nutrients (2). Similarly, Wnt/STOP decreases proteasomal degradation to facilitate cell size prior to mitosis in the cell cycle (19, 20, 101). Elucidating the molecular consequences of Wnt signalling effects on metabolism could offer novel insight into designing therapies that specifically target the Wnt-regulated metabolites.

### New Players in the Canonical Wnt Pathway for Tissue-Specific Therapies

The success of anti-sclerostin based antibody therapies for the treatment of low Wnt signalling in osteoporosis highlights the significance of identifying tissue specific Wnt regulators. The new crosstalk reported between classical Wnt signalling proteins and metabolic enzymes highlights a vast world of protein-protein interactions that have yet to be explored and may provide tissue-specific molecular targets.

**Targeted protein degradation (TPD)** is an emerging field that has the potential to revolutionize modern medicine (129, 130). These methods harness the endogenous degradation systems of the cell to target intracellular proteins for degradation. In the diseases driven by high Wnt signalling, the use of TPD approaches could be applied to degrade downstream cytosolic effectors of transcriptional Wnt programs. Altogether, canonical Wnt and the field of metabolic study is an open area of research that offers a unique opportunity to understand the fundamental basis of life and the cause of human disease.

## Author Contributions

CF and LA wrote the manuscript. MN, CF, and LA designed and created the figures. All authors contributed to the article and approved the submitted version.

## Conflict of Interest

The authors declare that the research was conducted in the absence of any commercial or financial relationships that could be construed as a potential conflict of interest.

## Publisher’s Note

All claims expressed in this article are solely those of the authors and do not necessarily represent those of their affiliated organizations, or those of the publisher, the editors and the reviewers. Any product that may be evaluated in this article, or claim that may be made by its manufacturer, is not guaranteed or endorsed by the publisher.
